# Brain-to-brain communication: the possible role of brain electromagnetic fields (As a Potential Hypothesis)

**DOI:** 10.1016/j.heliyon.2021.e06363

**Published:** 2021-03-01

**Authors:** Ehsan Hosseini

**Affiliations:** Division of Physiology, Department of Basic Sciences, Faculty of Veterinary Medicine, Urmia University, Urmia, Iran

**Keywords:** Brain to brain interface, Brain magnetic field, Cryptochrome, Brain magnetic particles, Brain subconscious centers, Mirror neuron

## Abstract

Up now, the communication between brains of different humans or animals has been confirmed and confined by the sensory medium and motor facilities of body. Recently, direct brain-to-brain communication (DBBC) outside the conventional five senses has been verified between animals and humans. Nevertheless, no empirical studies or serious discussion have been performed to elucidate the mechanism behind this process. The validation of DBBC has been documented via recording similar pattern of action potentials occurring in the brain cortex of two animals. With regard to action potentials in brain neurons, the magnetic field resulting from the action potentials created in neurons is one of the tools where the brain of one animal can affect the brain of another. It has been shown that different animals, even humans, have the power to understand the magnetic field. Cryptochrome, which exists in the retina and in different regions of the brain, has been confirmed to be able to perceive magnetic fields and convert magnetic fields to action potentials. Recently, iron particles (Fe_3_O_4_) believed to be functioning as magnets have been found in various parts of the brain, and are postulated as magnetic field receptors. Newly developed supersensitive magnetic sensors made of iron magnets that can sense the brain's magnetic field have suggested the idea that these Fe_3_O_4_ particles or magnets may be capable of perceiving the brain's extremely weak magnetic field. The present study suggests that it is possible the extremely week magnetic field in one animal's brain to transmit vital and accurate information to another animal's brain.

## 1 Introduction

Brain-to-brain communication, posited as one of the multiple kinds of telepathies, is the direct conveyance of feelings from one animal to another without using the common sensory channels of communication. In spite of many attempts to elucidate the mechanism of direct brain-to-brain communication between two animals and recording action potentials patterns occurring in the brain (Babiloni et al., 2006; King-Casas et al., 2005; Kingsbury et al., 2019; Liu and Pelowski, 2014), the molecular and cellular basis of this phenomenon is still unidentified. It is claimed that the ability to spot weak magnetic field energies may be a source of paranormal phenomena such as telepathy (M. A. Persinger and Healey, 2002). John Taylor and Eduardo Balanovski first discussed the electromagnetic fields emitted by human bodies as a potential mediator for telepathy (Taylor and Balanovski, 1979) and rejected this possibility according to the scientific evidence at that time. Their perception of the brain was as solely a physical body which ruled out biological processes and did not take into account the induction of action potentials (Roth and Basser, 1990) in the nerves or the existence of the protein Cryptochrome 2 as a supersensitive magnetic field receptor in the retina (Foley et al., 2011; Liedvogel and Mouritsen, 2010; Partch and Sancar, 2005) and numerous regions of the brain (Christiansen et al., 2016; Kim et al., 2018; Li et al., 2013; McCarthy and Welsh, 2012). Furthermore, they were unaware of the presence of magnetic particles in the brain (Gilder et al., 2018) which have been posited as magnetic field receptors (Shaw et al., 2015). According to McFadden, the brain's electromagnetic field (EMF) produces an image of the information in the neurons, and he claimed that brain's endogenous EMF impacts brain function (McFadden, 2002, 2013a) and proposed that the brain's EMF combines the information encrypted in millions of diverse neurons (McFadden, 2013b). Some evidence suggests that this theory may be correct (Agnati et al., 2018; Anastassiou et al., 2010; Fröhlich and McCormick, 2010; Reimann et al., 2013) demonstrating possibilities of a potential major role of EMF as a device of communication among cells inside the nervous system. In support of McFadden's hypothesis, researchers have attempted to decode human brain thoughts and emotions while recording electromagnetic activity in the cerebral cortex, thereby translating thoughts in the human brain into reading the brain as a text (Herff et al., 2015; Makin et al., 2019; Moses et al., 2019). This study indicates that electromagnetic fields can be meaningful and result from different thoughts and feelings formed in the brain. According to the theory of type physicalism (Boring, 1933; McLaughlin, 2010), different emotions include physical and chemical processes within a species which occurs among all its members in the same conformation. In accordance with the theory of type physicalism and research around translating the mind into readable text, it is possible that the text of the mind between different members of a species is intelligible to one another and is capable of transmitting through electromagnetic fields. In the recent years, with help of brain-machine interfaces, brain-to-brain interface has happened indirectly even between different species such as humans and animals (Gilja et al., 2015; Jarosiewicz et al., 2015; Nuyujukian et al., 2018). In human studies, telepathy has been weighed and confirmed (Dikker et al., 2017; Grau et al., 2014; Hildt, 2019; Jiang et al., 2019; Rao et al., 2014; Stephens et al., 2010). Also, brain-to-brain communication has evidently been validated in rats (Pais-Vieira et al., 2013; Tehovnik & Teixeira-e-Silva, 2014), Egyptian fruit bats (Zhang and Yartsev, 2019) and mice (Kingsbury et al., 2019). It has been proposed that the EM field contains uniform consciousness including feelings, insights, thoughts and emotions which can be felt and understood by every conscious creature in the world (Pockett, 2000). Considering all these points together, there is a possibility that such encrypted information as electromagnetic field packs can not only be transferred within one brain but also can be transferred from one brain to another and decoded as a pattern of action potentials, thus creating the same thoughts and emotions. The aim of this review is to shine a light on possible mechanisms of the cellular basis of the brain-to-brain interface in an attempt to open a novel field of behavioral studies focusing on potential functions of cryptochrome, magnetic particles and mirror neurons in extremely weak magnetic fields emitted by the brain.

## 2 Brain magnetic fields

It has been established that a magnetic field occurs in the brain, spreading around the brain, which is detectable by magnetoencephalography (MEG; magnetic field) method (Agnati et al., 2018). The key factor in producing brain electromagnetic fields is the action potential (Hales, 2014), a phenomenon that occurs in neurons resulting in membrane depolarization, with the departure of ions through the cell membrane (Cifra et al., 2011) producing an ion currents that is always associated with a magnetic field perpendicular to its direction according to the right-hand rule (Singh, 2014). The dendritic current of pyramidal neurons simultaneously firing in parallel is the basis of brain magnetic fields (Hämäläinen, 1991). One of the forms of action potential that happens in the brain is neural oscillation (Cebolla and Cheron, 2019). Neural oscillations are defined as repetitive patterns of action potentials occurring in neurons in the central nervous system (Başar, 2013). While oscillation related to a single neuron is intangible, the synchronized activity of a large numbers of neurons can give rise to large oscillations, generating a stronger EM field (CRICK, 1996), observable by MEG or EEG. To produce a measurable signal, almost 50,000 neurons are required to exert action potential together (Okada, 1983). It is stated that action potentials do not usually produce an effective field, mostly because the flows related to action potentials stream in opposite directions and the brain magnetic field does not exceed the power of 1000 femtotesla (10^−15^Tesla)) Magnetic field measurement unit is Tesla) (Pannetier et al., 2004). One study has shown that the main source of the magnetic field is in the limbic system region, including from septum to forebrain, as well encompassing the area from hypothalamus to ventral midbrain and possibly also including the hippocampus (Khan and Cohen, 2019).

## 3 Electromagnetic field detectors in the brain

According to Gregory Nordmann (Nordmann et al., 2017)**,** there are 3 prevailing proposals being weighed regarding how magnetic fields are sensed by animals. First is provocation of action potentials in neurons by electromagnetic induction; second is light-sensitive, chemical-based mechanism mediated by cryptochromes resulting in action potentials as nervous signals; and third is magnetite-based magnetoreceptors mechanically spotting the magnetic field, leading to neuronal action potentials.

### 3.1 Electromagnetic induction

**Figure 1:**
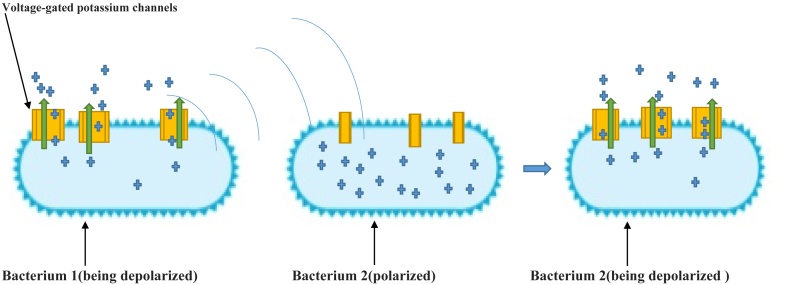
Depicting that magnetic field which is produced in a bacterium *Bacillus Subtilis* via potassium ions current that could affect the near bacterium and initiate action potential by induction law.  Representing pottasium positive ions,  Representing potasium voltage-gated ion channels,  Representing the current of pottasium ions to the outside of bacterium.  Indicating the transition of bacteria from polarization status to depolarization status. Action potentials in Bacillus Subtillus, described as Prindle, A., et al.(Prindle et al., 2015) are mediated by current of pottasium ions by potassium channels to the outside of the cell membrane. Polarized: The polarized status is defined as a state in which there is a difference in the concentration of an ion between the inside and outside of the membrane, resulting in a polarity or potential difference. Depolarized: The depolarized status is the state in which the polarity or potential difference between the outside and inside of the membrane is disappeared due to departure of relevant ion via membrane channels.

Various studies have found that microorganisms can convey information among themselves via electromagnetic fields (Cleaves and Thompson, 1936). For example, *Pseudomonas fluorescence*, a kind of bacteria, has been shown to communicate with one another using electromagnetic fields (Nikolaev et al., 2000) and it is indicated that *Escherichia coli* also has such ability (Trushin, 2003). A recently published paper found that membranes of bacteria depolarize via potassium ion channels and this depolarization propagates electrically to other bacteria by electrical stimulation just as happens between neurons (Prindle et al., 2015); while this paper did not discuss induction of action potentials between two bacteria which are close together, it may be surmised that this process could be very probable. In another recent study, the role of membrane-potential-based memory induced by potassium ion channels has been illustrated (Yang et al., 2020), reinforcing the possibility that the electromagnetic field created around the potassium channels is able to induce action potential in adjacent bacterial membranes, thus transferring the encoded memory to another bacterium (Figure 1). In the explanation of membrane-potential-based memory and also for more illustration of Figure 1, This can be explained that Current researches have indicated that memory can be encoded through targeted changes in the DNA sequence. This method has been effectively practical in numerous organizations, including bacteria, On the other hand, memory in biology is commonly associated with neurons in the brain, which use cellular membrane potential. Therefore, encoding memory at the membrane potential level in non-neuronal systems could offer novel insights to research memory creation and recovery. It has been revealed that bacteria undertaking membrane potential pulses are supposedly to experience them once more in the future, signifying that bacteria may have the capacity to store information about their past membrane potential state. This result proposes the likelihood that membrane potential-based memory could be encoded in a bacterial system.

Therefore, it may be supposed that encrypted cues such as memory can be transferred among bacteria by electromagnetic fields. Creating action potentials via electromagnetic induction resulting from a mass of neurons’ action potentials within a central nervous system has been asserted (Agnati et al., 2018) to act in such a way that the magnetic field due to the collective action potential of neurons induces action potential in the nearest neurons, thereby producing a neural message. This phenomenon is the topic of debate and is somewhat endorsed within the nervous system, but electromagnetic induction between two separate nervous systems has not yet been studied in practice or in theory.

A number of studies have indicated that exposure to extremely low frequency electromagnetic fields (ELF-EMF) modifies animal behaviors (Cui et al., 2012; Luukkonen et al., 2011; Piacentini et al., 2008). Although exposure to ELF-EMFs could be a factor in the development of anxious state or oxidative stress. It has been established that extremely low frequency magnetic fields with frequencies from 1-3 Hz provoke action potentials in large-diameter myelinated nerve axons (Reilly, 2012). Moreover, 50 Hz electromagnetic fields have empirically increased the frequency of action potentials in isolated nerves (Ebrahimian et al., 2013). Exposure to 50 Hz ELF-MF resulted in an increase in the peak amplitude of action potentials, and after hyperpolarization potential and magnetic fields decreased in a time-dependent manner as well as the firing frequency and the duration of the action potential (Moghadam et al., 2008). It was also revealed that the propagation of action potential along the membrane creates an inhomogeneous time-varying electromagnetic field (Isakovic et al., 2018). Furthermore, communication between neurons has been hypothesized and postulated by inhomogeneous electromagnetic fields produced and disseminated by neurons (Isakovic et al., 2018). The EMF field produced by just one neuron can reach spatially to hundreds of microns and can change the closer neurons' performance such as firing frequency (Gold et al., 2009). In this way, two neurons can communicate with together and affect each other's activity, and as a result, the electromagnetic fields derived from the firing of each neuron could be considered to be a new type of neurotransmitter. This phenomenon forms a fast message system between neurons called ephaptic coupling (Scholkmann, 2015) where synapses or gap junctions are not involved and are simply a result of a local electromagnetic field derived from a neuron (Hagen et al., 2016). Ephaptic coupling is facilitated the excitation of neurons (Katz and Schmitt, 1940) which means it can increases the speed of transmission of neural messages within the brain system and also from the environment to the brain and vice versa, and thus can increase the speed of cognition by the nervous system. Exposure to ELF-EMFs can improve recognition such as memory retention as in rats (Karimi et al., 2019). Also, ELF-MF has improving effect on different cognitive disorder signs of Alzheimer disease in rat (Akbarnejad et al., 2018) Furthermore, ELF-EMF improves social recognition memory in rats (Vázquez-García et al., 2004). As mentioned earlier that ephaptic coupling can increase the speed of cognition, It may be possible that a part of the improving effect of ELF-MF on recovery of cognition is medicated by the effect of ELF-MF on boosting ephaptic phenomena.

If a brain magnetic field as weak as of 10–1000 femtotesla (10^−15^Tesla) intensifies, is it possible that such magnetic fields can affect neuronal activity and trigger or change the pattern of action potentials of another brain via the electromagnetic induction rule? No studies currently exist on this topic, perhaps because magnetic fields with powers in the range of femtotesla (10^−15^Tesla) are incapable of inducing action potentials in neurons. Some studies have assayed the effect of extremely weak magnetic fields in the range of mT of power on physiological aspects of neuronal action potentials in such a way that pulsed magnetic field of 1–15 mT power induced synchronized neuronal firing of molluscan brain ganglia (Azanza, Calvo, & del Moral, 2002) or 0.8 mT intensity in a magnetic field could change the action potentials pattern of snail neurons (Moghadam et al., 2008).

## 4 Voltage-gated channels

Calcium fluxes are crucial detectors of EMF. On one hand, electromagnetic fields directly affect voltage-gated calcium channels and activate them (Pall, 2013, 2014, 2016), while on the other hand, voltage-gated calcium channel activation contributes to the release of neurotransmitters in the brain and also to the secretion of hormones by neuroendocrine cells (Berridge, 1998; Dunlap et al., 1995; Wheeler et al., 1994). In this way, the simultaneous large-scale firing of a large number of neurons can affect voltage-gated calcium channels in pre-synaptic neurons and stimulate them to increase the release of neurotransmitters. Therefore, voltage-gated calcium channels may be surmised as potential receptors of a magnetic field radiated by massive firing of neurons within one brain. As declared earlier, there are no studies to address if this possibility is viable between brain to brain. Studies have shown that voltage-gated sodium channels contribute to the rising phase of the neuronal action potential (Banasiak et al., 2004; Ding et al., 2008; Fraser et al., 2005). It is known that extremely low frequency electromagnetic fields such as brain waves are able to increase ion currents in sodium voltage-gated cerebellar granule cells (He et al., 2013) and magnetic field exposure changes ion channel function in neurons. It is possible that the organization of sodium channels are affected by magnetic fields (Zheng et al., 2017).

### 4.1 Cryptochrome

As sunlight is the main basis of energy on earth, primitive creatures such as archebacteria and cyanobacters have adapted to encounter and deal with the energy of sunlight, since these existed before the ozone layer. Cryptochromes appear to be absent from eubacteria and archaebacteria (Cashmore et al., 1999). Cyanobacters produce primitive cryptochromes to defend themselves from ultraviolet damage since they developed without the protective layer of ozone, the initial documented indications of the presence of cryptochromes (Sancar, 1994). Thus, the first documented indications of existence of primitive cryptochromes is in cyanobacteria that interface with the sunlight without the protective layer of ozone. Cyanobacteria have developed defenses, such as photolysis to repair ultraviolet-damaged DNA (Sancar, 1994). Cryptochromes have been found in various animal lineages, including insects, fish, amphibians, and mammals (Lin and Todo, 2005). The protein cryptochrome-2 that is expressed in the retina of vertebrates (Möller et al., 2004; Nießner et al., 2016; Thompson et al., 2003; Tosini et al., 2007; Tosini et al., 2008; Zhu and Green, 2001) exhibits the ability to detecting magnetic fields (Foley et al., 2011; Gegear et al., 2010; Yong, 2011). Activation of cryptochromes occurs in the presence of blue light (Foley et al., 2011; Gegear et al., 2008; Giachello et al., 2016) and a recent study in Arabidopsis established that cryptochromes detect magnetic field independently of light (Hammad et al., 2020). This latest finding could be the basis for the idea that cryptochromes can detect magnetic fields without depending on a specific wavelength of light; thus, cryptochromes in the retina and in different regions of brain may act similarly in detecting magnetic fields. It has been claimed that magnetic fields lead to ions flux unsteadiness in voltage-gated channels due to CRY-induced disruption of neuronal activity throughout the embryonic period of *Drosophila* (Giachello and Baines, 2015). Moreover, there is a report explicating that cryptochromes mediate neuronal excitation and increase action potential rates in *Drosophila* larvae (Marley et al., 2014). A study by Giachello et al. declared that magnetic fields modulate CRY activity and affect isolated neuron activity by increasing action potential firing (Giachello et al., 2016). The latter study proposes crucial proof to indicate that external magnetic fields are able to generate action potentials through alteration of the activity of cryptochromes and subsequently change animal behavior. It can be concluded that one of mediators by which magnetic fields affect brain neurons is through cryptochromes. CRY-mediated membrane depolarization initiated by a magnetic field is attributed to inactivation of potassium voltage-gated channels (Fogle et al., 2015); this establishes that cryptochromes are associated with voltage-gated channels and that an additional capability of cryptochromes is to generate action potentials under effect of a magnetic field. Cryptochrome-2 reacts to the earth's magnetic field at approximately 50μT power (Liedvogel and Mouritsen, 2010). The question that arises is whether the brain can create a magnetic field with the power of Earth's magnetic field. We know the magnitude of the magnetic field produced by a neuron is approximately 3.0 × 10 ^−12^ T (Isakovic et al., 2018). The cerebral cortex contains 100 billion neurons in human (Herculano-Houzel, 2009) and 21 billion neurons in rats (Korbo et al., 1990). Considering these points, if only one billion cerebral cortex neurons simultaneously activate in one direction, the magnetic field thus generated would be greater than the earth's magnetic field. The firing rates of cortical neurons are extremely variable in different tests (Faisal et al., 2008; Shadlen and Newsome, 1998). However, based on MEG measurements, the brain magnetic field is no greater than few hundred femtotesla (10^−15^Tesla) (Singh, 2014). Cortical fast-spiking neurons produce high-frequency action potentials reaching a frequency of 338Hz in the human temporal cortex, 450Hz in the frontal cortex of monkeys, and 215Hz in entorhinal cortexes (a part of the parahippocampal cortex) (B. Wang et al., 2016). Considering the number of these types of neurons and how many can fire together, it is theoretically possible to generate a magnetic field that can be understood or perceived by cryptochromes.

### 4.2 Magnetic particles

**Figure 2:**
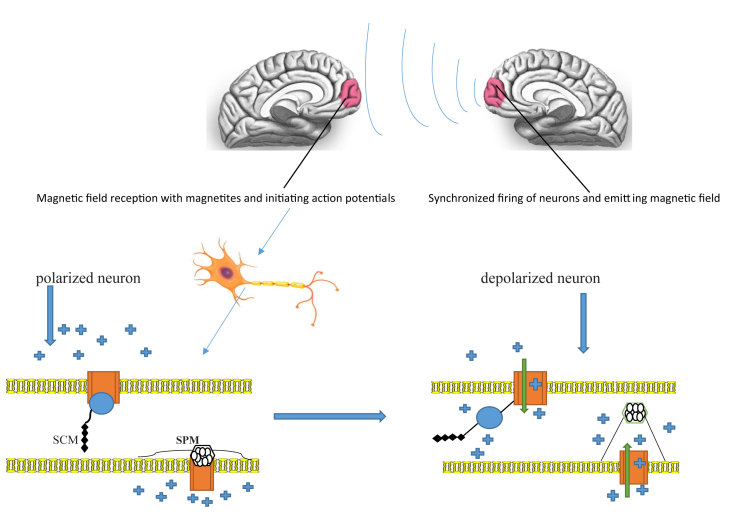
Exhibiting the reception of the magnetic field of a facing brain by magnetites which exist in prefrontal lobe of another brain and induction of action potentials in neurons. SPM (superparamagnetic magnetite), SCM (single chain magnetite),  Depicting positive ions chiefly sodium,  Depicting voltage-gated sodium channels,  Depicting the flux of sodium ions towards intracellular space.

Most living creatures need iron to exist; consequently, homeostasis of iron is crucial, and iron mineral collection may be the only method for a living creature to deposit surplus iron. Some species such as magnetotactic bacteria are capable of creating magnetite through oxide iron (Fe_3_O_4_) in the shape of crystal strands (Uebe and Schüler, 2016). The occurrence of such materials as chains of single-domain magnetite particles has been found in fish (J. Kirschvink, Walker, Chang, Dizon and Peterson, 1985), amphibians and reptiles (Perry et al., 1985), birds (R. Wiltschko and Wiltschko, 2013), and mammals such as rats (Barandiaran et al., 2015). The existence of magnetic particles (Fe_3_O_4_) in the human brain has been confirmed by various studies (J. L. Kirschvink, Kobayashi-Kirschvink and Woodford, 1992; Maher et al., 2016; Schultheiss-Grassi et al., 1999). It has also been established that the cerebral cortex, where similar action potential patterns have been recorded during brain-to-brain interface, contains magnetite similarly to other regions of the brain (Gilder et al., 2018). It has been suggested by some researchers that magnetic crystals are produced via internal biomineralization playing a physiological role (Kobayashi and Kirschvink, 1995). In contrast, others have proposed that magnetite in the brain may be derived from external sources and enter via the nasal sinus cavity and olfactory bulb (Maher et al., 2016). Magnetite is supposed to create a practical receptor for a magnetic field (J. L. Kirschvink, Walker and Diebel, 2001; Winklhofer and Kirschvink, 2010). It is assumed that there are two kind of magnetite, superparamagnetic (SPM) and single chain magnetite (SCM) (J. L. Kirschvink and Walker, 1985), which form the basis of a kind of magnetoreception. SPM has a small mass, making it incapable of keeping a steady magnetic moment, but it will line up in the path of an external magnetic field. SD magnetite is big enough to hold its own magnetic power. Various studies have attempted to focus on role of brain magnetite and cryptochromes in the earth's magnetic field navigation in birds (Cadiou and McNaughton, 2010; Heyers et al., 2007; W. Wiltschko, Munro, Wiltschko and Kirschvink, 2002), fishes (Hellinger and Hoffmann, 2012), turtles (Irwin and Lohmann, 2005), and these strictly ignore the extremely weak magnetic fields such that of the brain, which is in the range of 10–1000 femtotesla (10^−15^Tesla). However, one study has shown that magnetic fields with a magnitude of 20 femtotesla (10^−15^Tesla) can be detectable by instruments using nano-magnetite polymers, which consist of iron (Fe) and cobalt (Co) (Amirsolaimani et al., 2018). Another study found that it is possible to measure magnetic field of femtotesla (10^−15^Tesla) scale power using CoFe_2_O_4_ nanocrystals (Pavlopoulos et al., 2020). It has long been argued that the earth's magnetic waves can interfere with the measurement of the brain magnetic field and it has also been maintained that the magnetic field of the earth disturbs the ability to sense the brain's magnetic field. The two last-mentioned studies found that detection of a magnetic field in the range of the brain is possible without a need for a shield from the earth's magnetic field. These documents chiefly evoke the idea that the brain magnetites are potentially able to recognize dramatically weak magnetic field of brain that has not been discussed so far (Figure 2). Up to now, there has not been any empirical study which surveyed the exact mechanism by which these magnetites work to induce action potentials and cause an extremely weak magnetic field such as that of brain to be sensed.

## 5 Brain subconscious centers

**Figure 3:**
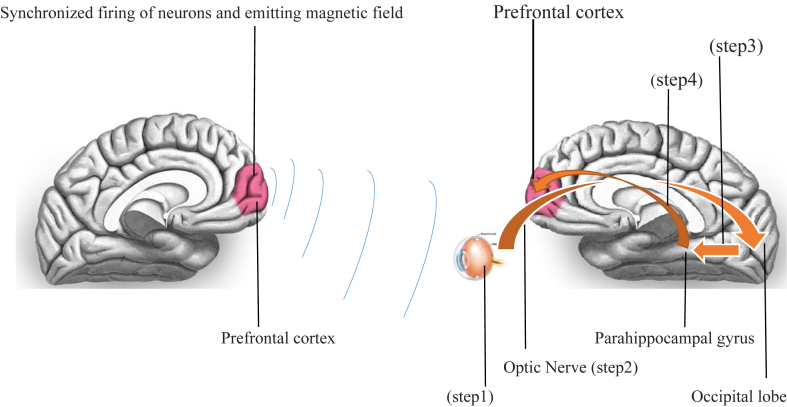
Indicating the brain to brain communication starting within retina via cryptochrome2 (step 1) that receives and processes magnetic field information then optic nerve sends this information to the occipital lobe (step 2), the region that detailed and classified information and then this information are sent to para-hippocampal gyrus (step 3) which additionally process and categorize information, specially in respect of emotions and thoughts and subsequently they are dispatched to prefrontal lobe (step 4) to be finally analyzed and concluded and make a propitious decision (step 1 is the reception of facing brain magnetic field by cryptochrome2, step 2 is sending processed information to the occipital lobe by optic nerve, step 3 is sending classified information from occipital lobe to para-hippocampal gyrus, step 4 is dispatching of detailed information from para-hippocampal gyrus to prefrontal lobe).

It is has long been supposed that animals subconsciously process magnetic fields such as the earth's magnetic field (C. X. Wang et al., 2019). One of the subconscious regions of the brain is the limbic system, encompassing amygdala, hippocampus, thalamus, hypothalamus, basal ganglia, and cingulate gyrus and parahippocampal gyrus (Solms, 2017). Using functional MRI, two studies reported considerable activation of the right parahippocampal gyrus after effective telepathy, indicating a basic role of the limbic system for brain-to-brain interface (M. A. Persinger and Saroka, 2012; Venkatasubramanian et al., 2008). A previous study revealed involvement of the hippocampal region in telepathy (Roll et al., 2002). These findings raise the question of whether the subconscious regions of the brain plays a central role in telepathy. The parahippocampal lobe plays a fundamental role in recognizing social situation (Schultz et al., 2010). Prominently, limbic regions associated with the hippocampus are imperative for empathy of the mind's conditions such as desires, intentions, and beliefs (Gorno-Tempini et al., 2004)**.** The parahippocampal cortex is associated with the frontal cortex (Aminoff et al., 2013; Goldman-Rakic et al., 1984; Suzuki, 2009). The frontal cortex includes the premotor cortex and the primary motor cortex – cortical parts of the motor cortex. The front part of the frontal lobe is covered by the prefrontal cortex. The primary motor cortex is one of the principal brain areas involved in motor function. The role of the primary motor cortex is to generate neural impulses that control the execution of movement, sending impulses to activate skeletal muscles for appropriate action. The prefrontal cortex is one of the major cortical afferents of the motor cortex by sending fiber comprising essential scheduling and execution (Yip and Lui, 2019). The prefrontal cortex is involved in the regulation of behavior (Dunn and Kronenberger, 2013) and collects information from various areas of the brain to process information to act appropriately to plan and reach objectives (Fuster, 2001; TEMBRA, 2018). In a recent study, it was shown that the occipital lobe was the brain region where a decoder person receives information sent from an encoder person (Jiang et al., 2019). Other studies established the same electroencephalographic records during telepathy in the occipital lobe (Duane and Behrendt, 1965; Kittenis et al., 2004; Standish et al., 2004; Wackermann et al., 2003) and concurrently in both the frontal and occipital lobes (M. Persinger, Koren and Tsang, 2003). Additional confirmation of involvement of the occipital lobe in telepathy has been proposed by functional magnetic resonance imaging (fMRI) studies (Moulton and Kosslyn, 2008; Richards et al., 2005; Standish et al., 2003). Another region of the brain involved in telepathy is the cuneus (Achterberg et al., 2005). The cuneus is a brain region associated with empathy (Jackson et al., 2006) and also with telepathy (M. Persinger, Roll et al., 2002; Roll et al., 2002). The cuneus is a smaller lobe within the occipital lobe of the brain. Interestingly, the occipital lobe includes the visual cortex, processing visual information partly sent by cryptochrome-2, a magnetic field sensitive protein, from the retina. It seems that the brain electromagnetic fields and collaborations among frontal cortex, occipital lobe and limbic system such as parahippocampal region could be a basis of this assertion as to how mammals such as rats, bats, and humans are capable of communication from brain to brain without any obvious signals (Figure 3).

## 6 Mirror neurons

Some neurons of the brain, mirror neurons, are triggered mutually when an animal acts and when the animal observes an equal action done by the other animal (dear Lafargue, 2014; Keysers, 2009). Such neurons have been spotted in primates (Rizzolatti et al., 1999), rats (Carrillo et al., 2019) and birds (Akins et al., 2002). In the human brain, the regions of the premotor cortex, the supplementary motor area, the primary somatosensory cortex and the inferior parietal cortex are confirmed to have mirror neurons (Adams, 2006) Mirror neurons seem to have a role in perceiving the actions of another animal, and in learning new skills by imitation (dear Lafargue, 2014). Mirror neurons are supposed to invoke actions which are observed (Schad, 2019). Furthermore, it is proposed that mirror neurons involve in the transfer of emotions, empathy, among animals (Gallese, 2001; Preston and De Waal, 2002). These neurons, contribute to the pre-conceptual and pre-verbal system of empathy, which means communication outside routine sense channels (Rizzolatti and Sinigaglia, 2008). It is hypothesized that telepathy phenomena, including thought transfer among humans, may be attributed to role of subconscious parts of brain mediated by mirror neurons (Haas, 2011), especially with regard to the existence of mirror neurons in subconscious centers of brain including cingulate amygdala, hippocampus, entorhinal cortex and parahippocampal gyrus (Lebedeva et al., 2019). Noticeably, as previously mentioned, the parahippocampal gyrus is one of the brain regions that may be involved in the brain to brain interface (M. A. Persinger and Saroka, 2012; Venkatasubramanian et al., 2008). It is believed that when mirror neurons fires, the Mu wave of the brain is suppressed (Pineda, 2005). It is also supposed that mirror neuros are involved in the design of intentions in the brain (Sinigaglia and Rizzolatti, 2011). Brain-computer interface (BCI) is a technology that transfers the thoughts and intentions of human to a computer by analyzing and processing the magnetic field of the brain; it especially has a lot of application in severely physically disabled persons such as paralytics. One type of BCI is suppression of the Mu wave of the brain, resulting in firing mirror neurons to help control and manage a computer to induce a desirable action on the part of a disabled person (Schomer and Da Silva, 2012). The question that may arise is whether we can replace a computer with a human brain. If so, we can conclude that electromagnetic waves emitted by synchronized action potentials of mirror neurons can play a prominent role in brain-to-brain communication especially in regard to transferring the intentions of one brain to another, just as happened in Pais-Vieira et al.'s study, where a second rat that was physically and visually completely separated from a first rat, pressed a lever that caused the pellet to fall on the shelf like the first rat (Pais-Vieira et al., 2013). We can presume the action of this rat was a kind of imitation, and mirror neurons are involved in imitation (Oztop et al., 2006). This could indicate why the role of mirror neurons are seriously argued in telepathy. More detailed studies are needed to clarify the role of the magnetic field produced by mirror neurons in brain-to-brain communication.

## 7 Conclusion

It may be hypothesized that large synchronized outbursts of cortex neurons in the frontal lobe, an area extensively involved in social cognition in a wide variety of mammalian species from rodents to primates (Adolphs, 2001; Amodio and Frith, 2006; Cao et al., 2018; Chang et al., 2013; Eliades and Miller, 2017; Forbes and Grafman, 2010; Liang et al., 2018; Miller, Thomas, Nummela and de la Mothe, 2015; Pais-Vieira et al., 2013), produces electromagnetic fields around the brain. This field may be able to influence cortical neurons in the frontal lobe of another brain by inducing action potentials in large groups of neurons which can transmit information such as different emotions and cognition cues to the other brain. A possible confirmation of this phenomenon is seen in two studies carried out, one in rats (Pais-Vieira et al., 2013) and one in Egyptian fruit bats (Zhang and Yartsev, 2019). In both studies, action potentials were recorded in the frontal cortexes of two rats or bats close to each other. Interestingly, after starting action potentials in animal 1 as the encoder, the same action potentials with the similiar patterns immediately appeared in the frontal cortex of animal 2 as the decoder. In the case of the rat study, the decoder rat also performed the same action as the first encoder rat (Pais-Vieira et al., 2013). The potential role of brain magnetic particles (magnetites) may be effective in perceiving another brain's magnetic field. It may be of significant interest in upcoming research to clarify the role of brain magnetic particles in brain-to-brain communication. The ubiquitous cryptochrome, a crucial receiver of the magnetic field, should be examined and its potential role in direct brain-to-brain communication may be elucidated by some fundamental research. The present study suggests that it is possible the extremely week magnetic fields of animal's brain could transmit vital and accurate information to another animal's brain.

## Declarations

### Author contribution statement

All authors listed have significantly contributed to the development and the writing of this article.

### Funding statement

This research did not receive any specific grant from funding agencies in the public, commercial, or not-for-profit sectors.

### Data availability statement

No data was used for the research described in the article.

### Declaration of interests statement

The authors declare no conflict of interest.

### Additional information

No additional information is available for this paper.

## References

[bib1] Achterberg J., Cooke K., Richards T., Standish L.J., Kozak L., Lake J. (2005). Evidence for correlations between distant intentionality and brain function in recipients: a functional magnetic resonance imaging analysis. J. Alternative Compl. Med.: Res. Paradigm Pract. Pol..

[bib2] Adams D.B. (2006). Brain mechanisms of aggressive behavior: an updated review. Neurosci. Biobehav. Rev..

[bib3] Adolphs R. (2001). The neurobiology of social cognition. Curr. Opin. Neurobiol..

[bib4] Agnati L.F., Guidolin D., Maura G., Marcoli M. (2018). Functional roles of three cues that provide nonsynaptic modes of communication in the brain: electromagnetic field, oxygen, and carbon dioxide. J. Neurophysiol..

[bib5] Akbarnejad Z., Esmaeilpour K., Shabani M., Asadi-Shekaari M., Saeedi goraghani M., Ahmadi-Zeidabadi M. (2018). Spatial memory recovery in Alzheimer's rat model by electromagnetic field exposure. Int. J. Neurosci..

[bib6] Akins C.K., Klein E.D., Zentall T.R. (2002). Imitative learning in Japanese quail (Coturnix japonica) using the bidirectional control procedure. Anim. Learn. Behav..

[bib7] Aminoff E.M., Kveraga K., Bar M. (2013). The role of the parahippocampal cortex in cognition. Trends Cognit. Sci..

[bib8] Amirsolaimani B., Gangopadhyay P., Persoons A.P., Showghi S.A., LaComb L.J., Norwood R.A., Peyghambarian N. (2018). High sensitivity magnetometer using nanocomposite polymers with large magneto-optic response. Optic Lett..

[bib9] Amodio D., Frith C. (2006). Meeting of minds: the medial frontal cortex and social cognition,. Nat. Rev. Neurosci..

[bib10] Anastassiou C.A., Montgomery S.M., Barahona M., Buzsáki G., Koch C. (2010). The effect of spatially inhomogeneous extracellular electric fields on neurons. J. Neurosci..

[bib11] Azanza M.J., Calvo A.C., del Moral A. (2002). Evidence of synchronization of neuronal activity of molluscan brain ganglia induced by alternating 50 Hz applied magnetic field. Electromagn. Biol. Med..

[bib12] Babiloni F., Cincotti F., Mattia D., Mattiocco M., Fallani F.D.V., Tocci A., Astolfi L. (2006). Hypermethods for EEG hyperscanning. Paper presented at the 2006 International Conference of the IEEE Engineering in Medicine and Biology Society.

[bib13] Banasiak K., Burenkova O., Haddad G. (2004). Activation of voltage-sensitive sodium channels during oxygen deprivation leads to apoptotic neuronal death. Neuroscience.

[bib14] Barandiaran J.M., Martinez-Millan L., Gerrikagoitia I., Orue S., Orue I., Lezama L., Fernández-Gubieda M.L. (2015). Search for magnetite nanoparticles in the rats’ brain. IEEE Trans. Magn..

[bib15] Başar E. (2013). Brain oscillations in neuropsychiatric disease. Dialogues Clin. Neurosci..

[bib16] Berridge M.J. (1998). Neuronal calcium signaling. Neuron.

[bib17] Boring E.G. (1933). The physical dimensions of consciousness.

[bib18] Cadiou H., McNaughton P.A. (2010). Avian magnetite-based magnetoreception: a physiologist's perspective. J. R. Soc. Interface.

[bib19] Cao W., Lin S., Xia Q.-q., Du Y.-l., Yang Q., Zhang M.-y., Xia J. (2018). Gamma oscillation dysfunction in mPFC leads to social deficits in neuroligin 3 R451C knockin mice. Neuron.

[bib20] Carrillo M., Han Y., Migliorati F., Liu M., Gazzola V., Keysers C. (2019). Emotional mirror neurons in the rat’s anterior cingulate cortex. Curr. Biol..

[bib21] Cashmore A.R., Jarillo J.A., Wu Y.-J., Liu D. (1999). Cryptochromes: blue light receptors for plants and animals. Science.

[bib22] Cebolla A.-M., Cheron G. (2019). Understanding neural oscillations in the human brain: from movement to consciousness and vice & versa. Front. Psychol..

[bib23] Chang S.W., Gariépy J.-F., Platt M.L. (2013). Neuronal reference frames for social decisions in primate frontal cortex. Nat. Neurosci..

[bib24] Christiansen S., Bouzinova E.V., Fahrenkrug J., Wiborg O. (2016). Altered expression pattern of clock genes in a rat model of depression. Int. J. Neuropsychopharmacol..

[bib25] Cifra M., Fields J.Z., Farhadi A. (2011). Electromagnetic cellular interactions. Prog. Biophys. Mol. Biol..

[bib26] Cleaves H., Thompson J. (1936). Invisible radiations of organisms. Nature.

[bib27] Crick F. (1996).

[bib28] Cui Y., Ge Z., Rizak J.D., Zhai C., Zhou Z., Gong S., Che Y. (2012). Deficits in water maze performance and oxidative stress in the hippocampus and striatum induced by extremely low frequency magnetic field exposure. PloS One.

[bib29] dear Lafargue M. (2014). Psyche and society.

[bib30] Dikker S., Wan L., Davidesco I., Kaggen L., Oostrik M., McClintock J., Ding M. (2017). Brain-to-brain synchrony tracks real-world dynamic group interactions in the classroom. Curr. Biol..

[bib31] Ding Y., Brackenbury W.J., Onganer P.U., Montano X., Porter L.M., Bates L.F., Djamgoz M.B. (2008). Epidermal growth factor upregulates motility of Mat-LyLu rat prostate cancer cells partially via voltage-gated Na+ channel activity. J. Cell. Physiol..

[bib32] Duane T.D., Behrendt T. (1965). Extrasensory electroencephalographic induction between identical twins. Science.

[bib33] Dunlap K., Luebke J.I., Turner T.J. (1995). Exocytotic Ca2+ channels in mammalian central neurons. Trends Neurosci..

[bib34] Dunn D.W., Kronenberger W.G. (2013).

[bib35] Ebrahimian H., Firoozabadi S.M., Janahmadi M., Kaviani Moghadam M. (2013). Parametric modeling of nerve cell under the sinusoidal environmental 50 Hz extremely low frequency magnetic fields. J. Ardabil Univ. Med. Sci..

[bib36] Eliades S.J., Miller C.T. (2017). Marmoset vocal communication: behavior and neurobiology. Dev. Neurobiol..

[bib37] Faisal A.A., Selen L.P., Wolpert D.M. (2008). Noise in the nervous system. Nat. Rev. Neurosci..

[bib38] Fogle K.J., Baik L.S., Houl J.H., Tran T.T., Roberts L., Dahm N.A., Holmes T.C. (2015). CRYPTOCHROME-mediated phototransduction by modulation of the potassium ion channel β-subunit redox sensor. Proc. Natl. Acad. Sci. Unit. States Am..

[bib39] Foley L.E., Gegear R.J., Reppert S.M. (2011). Human cryptochrome exhibits light-dependent magnetosensitivity. Nat. Commun..

[bib40] Forbes C.E., Grafman J. (2010). The role of the human prefrontal cortex in social cognition and moral judgment. Annu. Rev. Neurosci..

[bib41] Fraser S.P., Diss J.K., Chioni A.-M., Mycielska M.E., Pan H., Yamaci R.F., Grzywna Z. (2005). Voltage-gated sodium channel expression and potentiation of human breast cancer metastasis. Clin. Canc. Res..

[bib42] Fröhlich F., McCormick D.A. (2010). Endogenous electric fields may guide neocortical network activity. Neuron.

[bib43] Fuster J.M. (2001). The prefrontal cortex—an update: time is of the essence. Neuron.

[bib44] Gallese V. (2001). The'shared manifold'hypothesis. From mirror neurons to empathy. J. Conscious. Stud..

[bib45] Gegear R.J., Casselman A., Waddell S., Reppert S.M. (2008). Cryptochrome mediates light-dependent magnetosensitivity in Drosophila. Nature.

[bib46] Gegear R.J., Foley L.E., Casselman A., Reppert S.M. (2010). Animal cryptochromes mediate magnetoreception by an unconventional photochemical mechanism. Nature.

[bib47] Giachello C.N., Baines R.A. (2015). Inappropriate neural activity during a sensitive period in embryogenesis results in persistent seizure-like behavior. Curr. Biol..

[bib48] Giachello C.N., Scrutton N.S., Jones A.R., Baines R.A. (2016). Magnetic fields modulate blue-light-dependent regulation of neuronal firing by cryptochrome. J. Neurosci..

[bib49] Gilder S.A., Wack M., Kaub L., Roud S.C., Petersen N., Heinsen H., Schmitz C. (2018). Distribution of magnetic remanence carriers in the human brain. Sci. Rep..

[bib50] Gilja V., Pandarinath C., Blabe C.H., Nuyujukian P., Simeral J.D., Sarma A.A., Hochberg L.R. (2015). Clinical translation of a high-performance neural prosthesis. Nat. Med..

[bib51] Gold C., Girardin C.C., Martin K.A., Koch C. (2009). High-amplitude positive spikes recorded extracellularly in cat visual cortex. J. Neurophysiol..

[bib52] Goldman-Rakic P., Selemon L., Schwartz M. (1984). Dual pathways connecting the dorsolateral prefrontal cortex with the hippocampal formation and parahippocampal cortex in the rhesus monkey. Neuroscience.

[bib53] Gorno-Tempini M.L., Rankin K.P., Woolley J.D., Rosen H.J., Phengrasamy L., Miller B.L. (2004). Cognitive and behavioral profile in a case of right anterior temporal lobe neurodegeneration. Cortex: J. Devoted Stud. Nervous Syst. Behav..

[bib54] Grau C., Ginhoux R., Riera A., Nguyen T.L., Chauvat H., Berg M., Ruffini G. (2014). Conscious brain-to-brain communication in humans using non-invasive technologies. PloS One.

[bib55] Haas A.S. (2011). The interpretation of telepathy like effects: a novel electromagnetic and synchronistic version of the psychoanalytic model. Neuro Quantol..

[bib56] Hagen E., Dahmen D., Stavrinou M.L., Lindén H., Tetzlaff T., Van Albada S.J., Einevoll G.T. (2016). Hybrid scheme for modeling local field potentials from point-neuron networks. Cerebr. Cortex.

[bib57] Hales C. (2014). The origins of the brain's endogenous electromagnetic field and its relationship to provision of consciousness. J. Integr. Neurosci..

[bib58] Hämäläinen M. (1991). Basic principles of magnetoencephalography. Acta Radiologica.

[bib59] Hammad M., Albaqami M., Pooam M., Kernevez E., Witczak J., Ritz T., Ahmad M. (2020). Cryptochrome mediated magnetic sensitivity in Arabidopsis occurs independently of light-induced electron transfer to the flavin. Photochem. Photobiol. Sci..

[bib60] He Y.-L., Liu D.-D., Fang Y.-J., Zhan X.-Q., Yao J.-J., Mei Y.-A. (2013). Exposure to extremely low-frequency electromagnetic fields modulates Na+ currents in rat cerebellar granule cells through increase of AA/PGE2 and EP receptor-mediated cAMP/PKA pathway. PloS One.

[bib61] Hellinger J., Hoffmann K.-P. (2012). Magnetic field perception in the rainbow trout Oncorynchus mykiss: magnetite mediated, light dependent or both?. J. Comp. Physiol..

[bib62] Herculano-Houzel S. (2009). The human brain in numbers: a linearly scaled-up primate brain. Front. Hum. Neurosci..

[bib63] Herff C., Heger D., De Pesters A., Telaar D., Brunner P., Schalk G., Schultz T. (2015). Brain-to-text: decoding spoken phrases from phone representations in the brain. Front. Neurosci..

[bib64] Heyers D., Manns M., Luksch H., Güntürkün O., Mouritsen H. (2007). A visual pathway links brain structures active during magnetic compass orientation in migratory birds. PloS One.

[bib65] Hildt E. (2019). Multi-person brain-to-brain interfaces: ethical issues. Front. Neurosci..

[bib66] Irwin W.P., Lohmann K.J. (2005). Disruption of magnetic orientation in hatchling loggerhead sea turtles by pulsed magnetic fields. J. Comp. Physiol..

[bib67] Isakovic J., Dobbs-Dixon I., Chaudhury D., Mitrecic D. (2018). Modeling of inhomogeneous electromagnetic fields in the nervous system: a novel paradigm in understanding cell interactions, disease etiology and therapy. Sci. Rep..

[bib68] Jackson P.L., Brunet E., Meltzoff A.N., Decety J. (2006). Empathy examined through the neural mechanisms involved in imagining how I feel versus how you feel pain. Neuropsychologia.

[bib69] Jarosiewicz B., Sarma A.A., Bacher D., Masse N.Y., Simeral J.D., Sorice B., Gilja V. (2015). Virtual typing by people with tetraplegia using a self-calibrating intracortical brain-computer interface. Sci. Transl. Med..

[bib70] Jiang L., Stocco A., Losey D.M., Abernethy J.A., Prat C.S., Rao R.P. (2019). BrainNet: a multi-person brain-to-brain interface for direct collaboration between brains. Sci. Rep..

[bib71] Karimi S.A., Salehi I., Shykhi T., Zare S., Komaki A. (2019). Effects of exposure to extremely low-frequency electromagnetic fields on spatial and passive avoidance learning and memory, anxiety-like behavior and oxidative stress in male rats. Behav. Brain Res..

[bib72] Katz B., Schmitt O.H. (1940). Electric interaction between two adjacent nerve fibres. J. Physiol..

[bib73] Keysers C. (2009). Mirror neurons. Curr. Biol..

[bib74] Khan S., Cohen D. (2019). Using the magnetoencephalogram to noninvasively measure magnetite in the living human brain. Hum. Brain Mapp..

[bib75] Kim S.H., Park H.G., Jeong S.H., Kang U.G., Ahn Y.M., Kim Y.S. (2018). Electroconvulsive seizure alters the expression and daily oscillation of circadian genes in the rat frontal cortex. Psychiatr. Investig..

[bib76] King-Casas B., Tomlin D., Anen C., Camerer C.F., Quartz S.R., Montague P.R. (2005). Getting to know you: reputation and trust in a two-person economic exchange. Science.

[bib77] Kingsbury L., Huang S., Wang J., Gu K., Golshani P., Wu Y.E., Hong W. (2019). Correlated neural activity and encoding of behavior across brains of socially interacting animals. Cell.

[bib78] Kirschvink J., Walker M., Chang S.-B., Dizon A., Peterson K. (1985). Chains of single-domain magnetite particles in chinook salmon, Oncorhynchus tshawytscha. J. Comp. Physiol..

[bib79] Kirschvink J.L., Kobayashi-Kirschvink A., Woodford B.J. (1992). Magnetite biomineralization in the human brain. Proc. Natl. Acad. Sci. Unit. States Am..

[bib80] Kirschvink J.L., Walker M.M. (1985). Particle-size considerations for magnetite-based magnetoreceptors. Magnetite Biomineralization and Magnetoreception in Organisms.

[bib81] Kirschvink J.L., Walker M.M., Diebel C.E. (2001). Magnetite-based magnetoreception. Curr. Opin. Neurobiol..

[bib82] Kittenis M., Caryl P., Stevens P. (2004). Distant psychophysiological interaction effects between related and unrelated participants. Paper Presented at the Proceedings of the Parapsychological Association Convention.

[bib83] Kobayashi A., Kirschvink J.L. (1995). Magnetoreception and electromagnetic field effects: sensory perception of the geomagnetic field in animals and humans.

[bib84] Korbo L., Pakkenberg B., Ladefoged O., Gundersen H.J.G., Arlien-Søborg P., Pakkenberg H. (1990). An efficient method for estimating the total number of neurons in rat brain cortex. J. Neurosci. Methods.

[bib85] Lebedeva N., Karimova E., Karpichev V., Maltsev V.Y. (2019). The mirror system of the brain on observation, performance, and imagination of motor tasks–neurophysiological reflection of the perception of another Person’s consciousness. Neurosci. Behav. Physiol..

[bib86] Li J.Z., Bunney B.G., Meng F., Hagenauer M.H., Walsh D.M., Vawter M.P., Barchas J.D. (2013). Circadian patterns of gene expression in the human brain and disruption in major depressive disorder. Proc. Natl. Acad. Sci. Unit. States Am..

[bib87] Liang B., Zhang L., Barbera G., Fang W., Zhang J., Chen X., Lin D.-T. (2018). Distinct and dynamic ON and OFF neural ensembles in the prefrontal cortex code social exploration. Neuron.

[bib88] Liedvogel M., Mouritsen H. (2010). Cryptochromes—a potential magnetoreceptor: what do we know and what do we want to know? Journal of the Royal Society Interface.

[bib89] Lin C., Todo T. (2005). The cryptochromes. Genome Biol..

[bib90] Liu T., Pelowski M. (2014). A new research trend in social neuroscience: towards an interactive-brain neuroscience. PsyCh J..

[bib91] Luukkonen J., Liimatainen A., Höytö A., Juutilainen J., Naarala J. (2011). Pre-exposure to 50 Hz magnetic fields modifies menadione-induced genotoxic effects in human SH-SY5Y neuroblastoma cells. PloS One.

[bib92] Maher B.A., Ahmed I.A., Karloukovski V., MacLaren D.A., Foulds P.G., Allsop D., Calderon-Garciduenas L. (2016). Magnetite pollution nanoparticles in the human brain. Proc. Natl. Acad. Sci. Unit. States Am..

[bib93] Makin J.G., Moses D.A., Chang E.F. (2019). Machine translation of cortical activity to text with an encoder-decoder framework. bioRxiv.

[bib94] Marley R., Giachello C.N., Scrutton N.S., Baines R.A., Jones A.R. (2014). Cryptochrome-dependent magnetic field effect on seizure response in Drosophila larvae. Sci. Rep..

[bib95] McCarthy M.J., Welsh D.K. (2012). Cellular circadian clocks in mood disorders. J. Biol. Rhythm..

[bib96] McFadden J. (2002). Synchronous Firing and its influence on the brain's electromagnetic field. J. Conscious. Stud..

[bib97] McFadden J. (2013). The CEMI field theory closing the loop. J. Conscious. Stud..

[bib98] McFadden J. (2013). The CEMI field theory gestalt information and the meaning of meaning.

[bib99] McLaughlin B.P. (2010). Consciousness, type physicalism, and inference to the best explanation. Phil. Issues.

[bib100] Miller C.T., Thomas A.W., Nummela S.U., de la Mothe L.A. (2015). Responses of primate frontal cortex neurons during natural vocal communication. J. Neurophysiol..

[bib101] Moghadam M.K., Firoozabadi S.M., Janahmadi M. (2008). 50 Hz alternating extremely low frequency magnetic fields affect excitability, firing and action potential shape through interaction with ionic channels in snail neurones. Environmentalist.

[bib102] Möller A., Sagasser S., Wiltschko W., Schierwater B. (2004). Retinal cryptochrome in a migratory passerine bird: a possible transducer for the avian magnetic compass. Naturwissenschaften.

[bib103] Moses D.A., Leonard M.K., Makin J.G., Chang E.F. (2019). Real-time decoding of question-and-answer speech dialogue using human cortical activity. Nat. Commun..

[bib104] Moulton S.T., Kosslyn S.M. (2008). Using neuroimaging to resolve the psi debate. J. Cognit. Neurosci..

[bib105] Nießner C., Denzau S., Malkemper E.P., Gross J.C., Burda H., Winklhofer M., Peichl L. (2016). Cryptochrome 1 in retinal cone photoreceptors suggests a novel functional role in mammals. Sci. Rep..

[bib106] Nikolaev Y.A., Prosser J., Wheatley R. (2000). Regulation of the adhesion of Pseudomonas fluorescens cells to glass by extracellular volatile compounds. Microbiology.

[bib107] Nordmann G.C., Hochstoeger T., Keays D.A. (2017). Unsolved mysteries: magnetoreception—A sense without a receptor. PLoS Biol..

[bib108] Nuyujukian P., Sanabria J.A., Saab J., Pandarinath C., Jarosiewicz B., Blabe C.H., Simeral J.D. (2018). Cortical control of a tablet computer by people with paralysis. PloS One.

[bib109] Okada Y. (1983). Biomagnetism: Neurogenesis of Evoked Magnetic fields.

[bib110] Oztop E., Kawato M., Arbib M. (2006). Mirror neurons and imitation: a computationally guided review. Neural Network..

[bib111] Pais-Vieira M., Lebedev M., Kunicki C., Wang J., Nicolelis M.A. (2013). A brain-to-brain interface for real-time sharing of sensorimotor information. Sci. Rep..

[bib112] Pall M.L. (2013). Electromagnetic fields act via activation of voltage-gated calcium channels to produce beneficial or adverse effects. J. Cell Mol. Med..

[bib113] Pall M.L. (2014). Electromagnetic field activation of voltage-gated calcium channels: role in therapeutic effects. Electromagn. Biol. Med..

[bib114] Pall M.L. (2016). Microwave frequency electromagnetic fields (EMFs) produce widespread neuropsychiatric effects including depression. J. Chem. Neuroanat..

[bib115] Pannetier M., Fermon C., Le Goff G., Simola J., Kerr E. (2004). Femtotesla magnetic field measurement with magnetoresistive sensors. Science.

[bib116] Partch C.L., Sancar A. (2005). Cryptochromes and circadian photoreception in animals.

[bib117] Pavlopoulos N.G., Kang K., Holmen L., Lyons N., Akhoundi F., Carothers K., Phan A. (2020). Polymer and magnetic nanoparticle composites with tunable magneto-optical activity: role of nanoparticle dispersion for high verdet constant materials. J. Mater. Chem. C.

[bib118] Perry A., Bauer G.B., Dizon A.E. (1985). Magnetoreception and biomineralization of magnetite in amphibians and reptiles. Magnetite Biomineralization and Magnetoreception in Organisms.

[bib119] Persinger M., Koren S., Tsang E. (2003). Enhanced power within a specific band of theta activity in one person while another receives circumcerebral pulsed magnetic fields: a mechanism for cognitive influence at a distance?. Percept. Mot. Skills.

[bib120] Persinger M., Roll W., Tiller S., Koren S., Cook C. (2002). Remote viewing with the artist Ingo Swann: neuropsychological profile, electroencephalographic correlates, magnetic resonance imaging (MRI), and possible mechanisms. Percept. Mot. Skills.

[bib121] Persinger M.A., Healey F. (2002). Experimental facilitation of the sensed presence: possible intercalation between the hemispheres induced by complex magnetic fields. J. Nerv. Ment. Dis..

[bib122] Persinger M.A., Saroka K.S. (2012). Protracted parahippocampal activity associated with Sean Harribance. Int. J. Yoga.

[bib123] Piacentini R., Ripoli C., Mezzogori D., Azzena G.B., Grassi C. (2008). Extremely low-frequency electromagnetic fields promote in vitro neurogenesis via upregulation of Cav1-channel activity. J. Cell. Physiol..

[bib124] Pineda J.A. (2005). The functional significance of mu rhythms: translating “seeing” and “hearing” into “doing”. Brain Res. Rev..

[bib125] Pockett S. (2000). The nature of consciousness: a hypothesis: IUniverse.

[bib126] Preston S.D., De Waal F.B. (2002). Empathy: its ultimate and proximate bases. Behav. Brain Sci..

[bib127] Prindle A., Liu J., Asally M., Ly S., Garcia-Ojalvo J., Süel G.M. (2015). Ion channels enable electrical communication in bacterial communities. Nature.

[bib128] Rao R.P., Stocco A., Bryan M., Sarma D., Youngquist T.M., Wu J., Prat C.S. (2014). A direct brain-to-brain interface in humans. PloS One.

[bib129] Reilly J.P. (2012). Applied bioelectricity: from electrical stimulation to electropathology: springer science & business media.

[bib130] Reimann M.W., Anastassiou C.A., Perin R., Hill S.L., Markram H., Koch C. (2013). A biophysically detailed model of neocortical local field potentials predicts the critical role of active membrane currents. Neuron.

[bib131] Richards T.L., Kozak L., Johnson L.C., Standish L.J. (2005). Replicable functional magnetic resonance imaging evidence of correlated brain signals between physically and sensory isolated subjects. J. Alternative Compl. Med.: Res. Paradigm Pract. Pol..

[bib132] Rizzolatti G., Fadiga L., Fogassi L., Gallese V. (1999). Resonance behaviors and mirror neurons. Arch. Ital. Biol..

[bib133] Rizzolatti G., Sinigaglia C. (2008). Mirrors in the Brain: How Our Minds Share Actions and Emotions.

[bib134] Roll W., Persinger M., Webster D., Tiller S., Cook C. (2002). Neurobehavioral and neurometabolic (SPECT) correlates of paranormal information: involvement of the right hemisphere and its sensitivity to weak complex magnetic fields. Int. J. Neurosci..

[bib135] Roth B.J., Basser P.J. (1990). A model of the stimulation of a nerve fiber by electromagnetic induction. IEEE (Inst. Electr. Electron. Eng.) Trans. Biomed. Eng..

[bib136] Sancar A. (1994). Structure and function of DNA photolyase. Biochemistry.

[bib137] Schad J.N. (2019). Mirror neuron; A beautiful unnecessary concept. arXiv.

[bib138] Scholkmann F. (2015). Two emerging topics regarding long-range physical signaling in neurosystems: membrane nanotubes and electromagnetic fields. J. Integr. Neurosci..

[bib139] Schomer D.L., Da Silva F.L. (2012). Niedermeyer's Electroencephalography: Basic Principles, Clinical Applications, and Related fields.

[bib140] Schultheiss-Grassi P.P., Wessiken R., Dobson J. (1999). TEM investigations of biogenic magnetite extracted from the human hippocampus. Biochim. Biophys. Acta Gen. Subj..

[bib141] Schultz C.C., Koch K., Wagner G., Roebel M., Nenadic I., Gaser C., Schlösser R.G. (2010). Increased parahippocampal and lingual gyrification in first-episode schizophrenia. Schizophr. Res..

[bib142] Shadlen M.N., Newsome W.T. (1998). The variable discharge of cortical neurons: implications for connectivity, computation, and information coding. J. Neurosci..

[bib143] Shaw J., Boyd A., House M., Woodward R., Mathes F., Cowin G., Baer B. (2015). Magnetic particle-mediated magnetoreception. J. R. Soc. Interface.

[bib144] Singh S.P. (2014). Magnetoencephalography: basic principles. Ann. Indian Acad. Neurol..

[bib145] Sinigaglia C., Rizzolatti G. (2011). Through the looking glass: self and others. Conscious. Cognit..

[bib146] Solms M. (2017). What is “the unconscious,” and where is it located in the brain? A neuropsychoanalytic perspective. Ann. NY Acad. Sci..

[bib147] Standish L.J., Johnson L.C., Kozak L., Richards T. (2003). Evidence of correlated functional magnetic resonance imaging signals between distant human brains. Alternative Ther. Health Med..

[bib148] Standish L.J., Kozak L., Johnson L.C., Richards T. (2004). Electroencephalographic evidence of correlated event-related signals between the brains of spatially and sensory isolated human subjects. J. Alternative Compl. Med..

[bib149] Stephens G.J., Silbert L.J., Hasson U. (2010). Speaker–listener neural coupling underlies successful communication. Proc. Natl. Acad. Sci. Unit. States Am..

[bib150] Suzuki W.A. (2009). Comparative analysis of the cortical afferents, intrinsic projections, and interconnections of the parahippocampal region in monkeys and rats.

[bib151] Taylor J., Balanovski E. (1979). Is there any scientific explanation of the paranormal?. Nature.

[bib152] Tehovnik E., Teixeira-e-Silva Z. (2014). Brain-to-brain interface for real-time sharing of sensorimotor information: a commentary. OA Neurosci..

[bib153] Tembra D.P.D.S. (2018). Efeitos do exercício físico sore parâmetros cognitivos e bioquímicos em ratos expostos ao ethanol de forma intensa e episódica (Binge Drinking).

[bib154] Thompson C.L., Rickman C.B., Shaw S.J., Ebright J.N., Kelly U., Sancar A., Rickman D.W. (2003). Expression of the blue-light receptor cryptochrome in the human retina. Invest. Ophthalmol. Vis. Sci..

[bib155] Tosini G., Kasamatsu M., Sakamoto K. (2007). Clock gene expression in the rat retina: effects of lighting conditions and photoreceptor degeneration. Brain Res..

[bib156] Tosini G., Pozdeyev N., Sakamoto K., Iuvone P.M. (2008). The circadian clock system in the mammalian retina. Bioessays.

[bib157] Trushin M.V. (2003). Studies on distant regulation of bacterial growth and light emission. Microbiology.

[bib158] Uebe R., Schüler D. (2016). Magnetosome biogenesis in magnetotactic bacteria. Nat. Rev. Microbiol..

[bib159] Vázquez-García M., Elías-Viñas D., Reyes-Guerrero G., Domínguez-González A., Verdugo-Díaz L., Guevara-Guzmán R. (2004). Exposure to extremely low-frequency electromagnetic fields improves social recognition in male rats. Physiol. Behav..

[bib160] Venkatasubramanian G., Jayakumar P.N., Nagendra H.R., Nagaraja D., Deeptha R., Gangadhar B.N. (2008). Investigating paranormal phenomena: functional brain imaging of telepathy. Int. J. Yoga.

[bib161] Wackermann J., Seiter C., Keibel H., Walach H. (2003). Correlations between brain electrical activities of two spatially separated human subjects. Neurosci. Lett..

[bib162] Wang B., Ke W., Guang J., Chen G., Yin L., Deng S., Zheng R. (2016). Firing frequency maxima of fast-spiking neurons in human, monkey, and mouse neocortex. Front. Cell. Neurosci..

[bib163] Wang C.X., Hilburn I.A., Wu D.-A., Mizuhara Y., Cousté C.P., Abrahams J.N., Kirschvink J.L. (2019). Transduction of the geomagnetic field as evidenced from alpha-band activity in the human brain. Eneuro.

[bib164] Wheeler D.B., Randall A., Tsien R.W. (1994). Roles of N-type and Q-type Ca2+ channels in supporting hippocampal synaptic transmission. Science.

[bib165] Wiltschko R., Wiltschko W. (2013). The magnetite-based receptors in the beak of birds and their role in avian navigation. J. Comp. Physiol..

[bib166] Wiltschko W., Munro U., Wiltschko R., Kirschvink J.L. (2002). Magnetite-based magnetoreception in birds: the effect of a biasing field and a pulse on migratory behavior. J. Exp. Biol..

[bib167] Winklhofer M., Kirschvink J.L. (2010). A quantitative assessment of torque-transducer models for magnetoreception. J. R. Soc. Interface.

[bib168] Yang C.-Y., Bialecka-Fornal M., Weatherwax C., Larkin J., Prindle A., Liu J., Suel G.M. (2020). Encoding spatial memory within a bacterial biofilm community. Biophys. J..

[bib169] Yip D.W., Lui F. (2019). Physiology, motor cortical. StatPearls [Internet]: StatPearls Publishing.

[bib170] Yong E. (2011). Humans have a magnetic sensor in our eyes, but can we detect magnetic fields?. DiscoverMagazine.com.

[bib171] Zhang W., Yartsev M.M. (2019). Correlated neural activity across the brains of socially interacting bats. Cell.

[bib172] Zheng Y., Dou J.-r., Gao Y., Dong L., Li G. (2017). Effects of 15 Hz square wave magnetic fields on the voltage-gated sodium and potassium channels in prefrontal cortex pyramidal neurons. Int. J. Radiat. Biol..

[bib173] Zhu H., Green C.B. (2001). Three cryptochromes are rhythmically expressed in Xenopus laevis retinal photoreceptors. Mol. Vis..

